# Decompressive hemicraniectomy for acute ischemic stroke in a patient implanted with a left ventricular assist device: a case report

**DOI:** 10.1186/s12872-020-01576-0

**Published:** 2020-06-10

**Authors:** Walid Oulehri, Mircea Cristinar, Gharib Ajob, Sandrine Marguerite, Bob Heger, Hélène Cebula, Michel Kindo, Paul Michel Mertes

**Affiliations:** 1 Pôle Anesthésie Réanimation Chirurgicale, service de Réanimation Chirurgicale, Hôpitaux Universitaires de Strasbourg NHC, 1, Place de l’Hôpital, 67000 Strasbourg, France; 2 EA 3072, Fédération de Médecine Translationnelle de Strasbourg, Institut de Physiologie, Université de Strasbourg, 67000 Strasbourg, France; 3 Pôle Tête et Cou, service de Neurochirurgie, Hôpitaux Universitaires de Strasbourg NHC, 67000 Strasbourg, France; 4 Pôle Cardiologie, service de Chirurgie Cardiovasculaire, Hôpitaux Universitaires de Strasbourg NHC, 67000 Strasbourg, France

**Keywords:** Decompressive craniectomy, Cardiac arrest, Left ventricular assist device, Ischemic stroke, Anticoagulation, Heart transplantation

## Abstract

**Background:**

Thromboembolic ischemic stroke (IS) is one of the most feared complications of left ventricular assist device (LVAD) placement and represents a challenge to surgical management because of concomitant anticoagulant therapy.

**Case presentation:**

A 39-year-old man presented with cardiogenic shock following an out-of-hospital cardiac arrest. After a period of stabilization, the patient was referred for LVAD placement. Upon recovery from anesthesia, he presented with acute neurological deficits suggestive of IS. A brain computed tomography confirmed the diagnosis, and an emergency decompressive hemicraniectomy (DHC) was performed. Anticoagulation was managed empirically. The patient’s neurological status progressively improved and he was referred for heart transplantation at five months from DHC. One month later, cranioplasty was performed.

**Conclusions:**

This report suggests an anticoagulation management approach in combination with decompressive craniectomy after IS in a patient with LVAD placement was successful. An optimized anticoagulation management and collaborative team-based practice may contribute to successful outcomes in complex cases.

## Background

Heart failure (HF) is a major public health concern resulting in substantial morbidity, mortality, and healthcare expenditures worldwide. Heart transplantation (HT) remains the treatment of choice for improving survival in patients suffering from advanced HF, although organ shortage restricts this option. In this scenario, a left ventricular assist device (LVAD) can be implanted to bridge patients to HT. The optimal anticoagulation strategy to avoid thrombotic complications (e.g., pump thrombosis and stroke) in patients with LVADs remains an ongoing challenge especially in complex clinical situations. To our knowledge, there is still no consensus on how to best manage patients with LVADs who require emergency decompressive hemicraniectomy (DHC) following a malignant ischemic stroke (IS). In the present case report, we describe how an optimized anticoagulation management and collaborative team-based practice are paramount to prevent catastrophic outcomes in such challenging cases.

## Case presentation

**Fig. 1:**
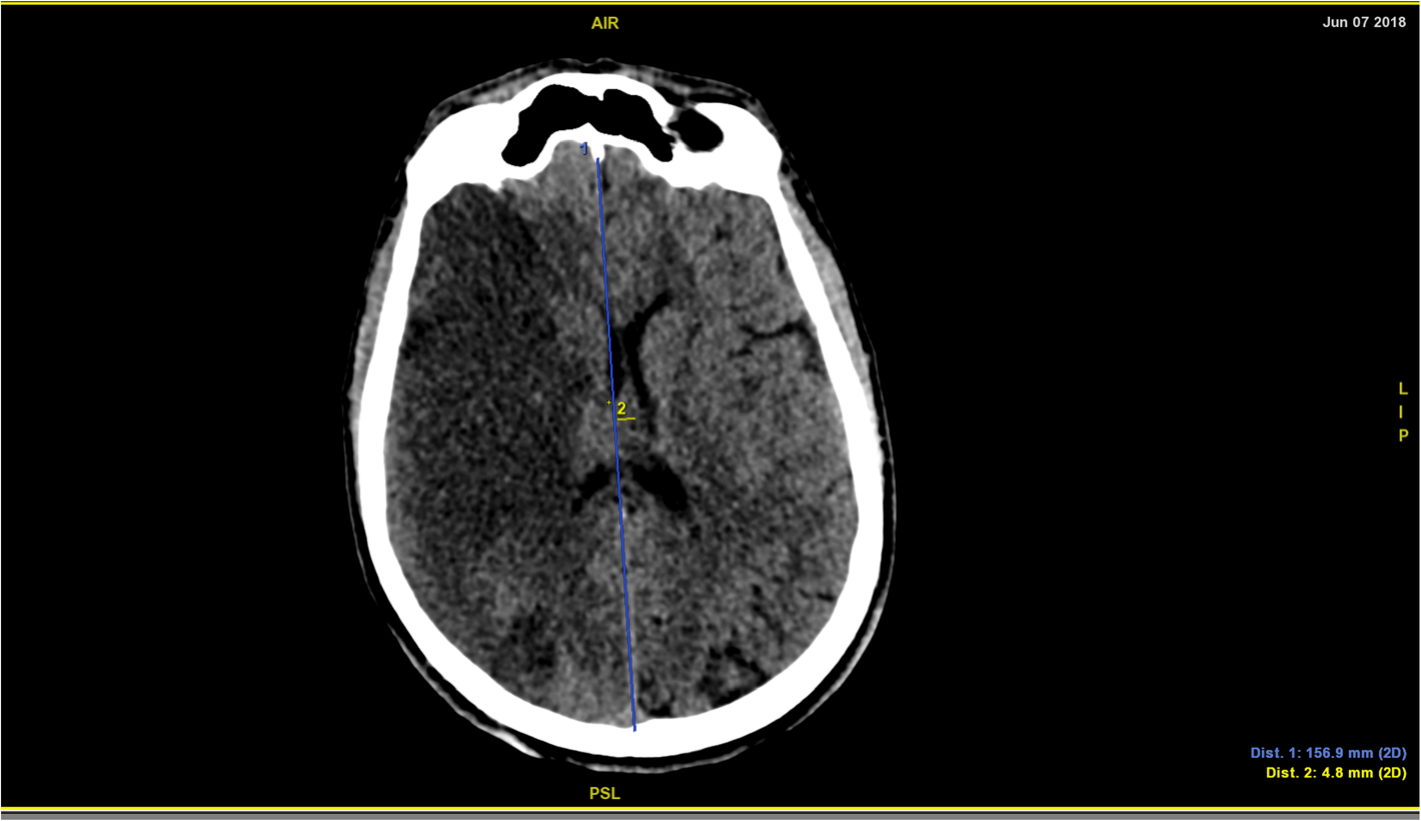
A brain computed tomography scan without contrast revealed a right sylvian ischemic stroke with a mass effect and a midline shift of 4.8 mm

**Fig. 2:**
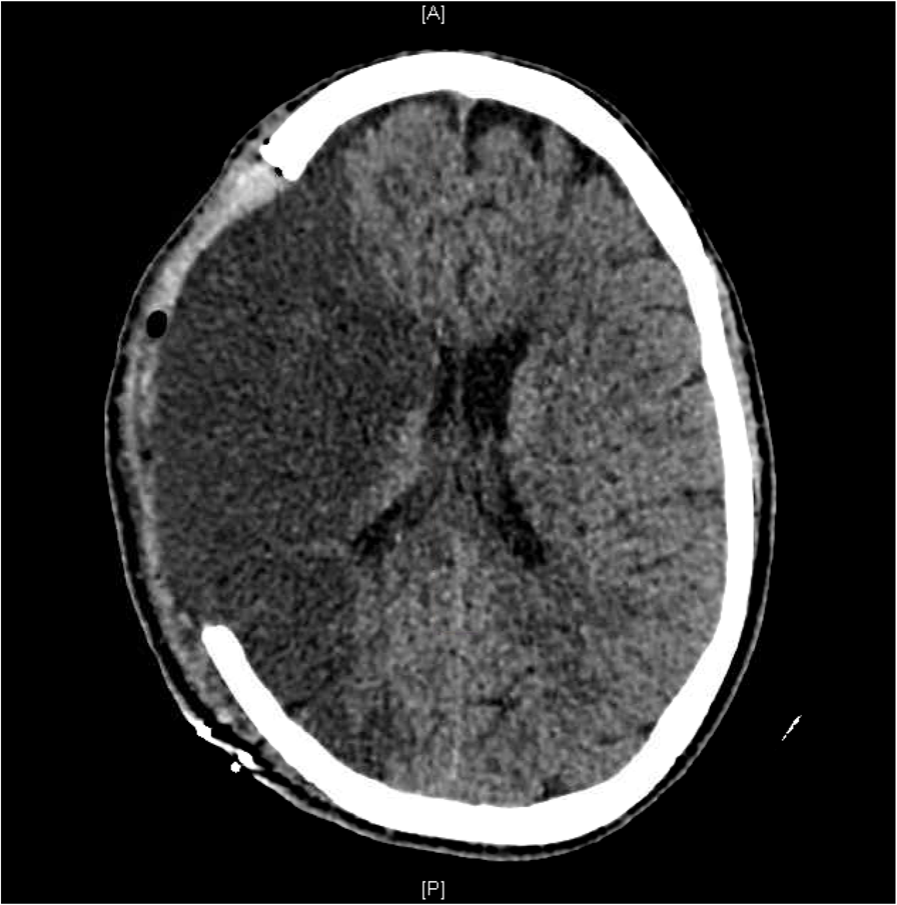
A brain computed tomography scan illustrating right decompressive hemicraniectomy

**Fig. 3:**
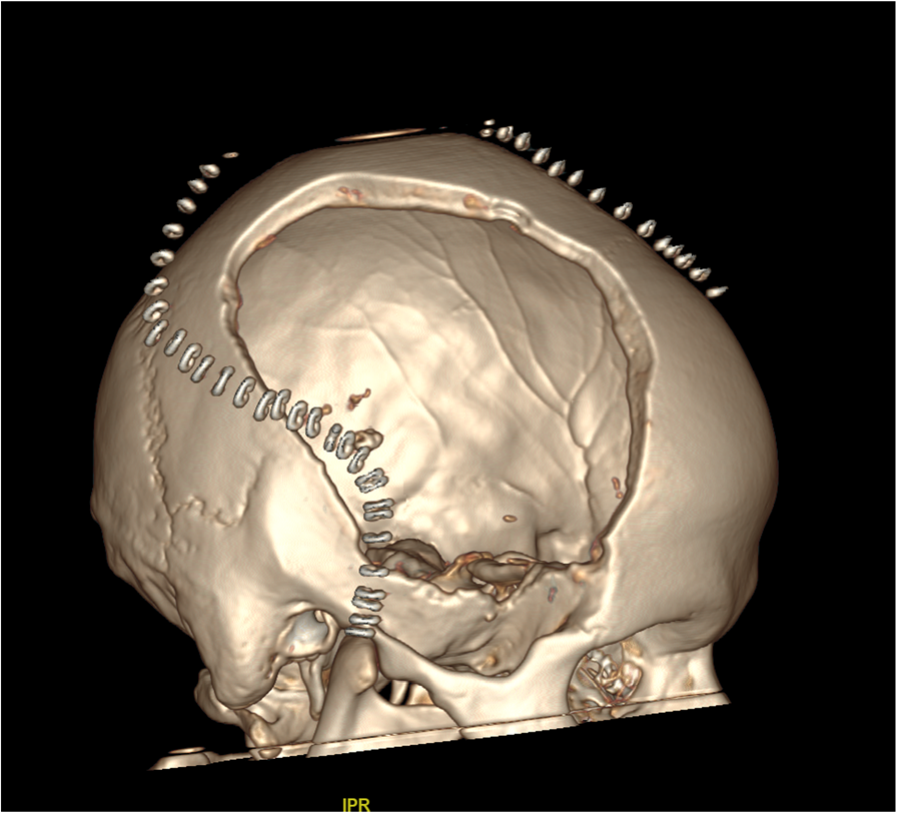
Three-dimensional rendering of the right decompressive hemicraniectomy

A 39-year-old man – with a negative personal history for cardiovascular disorders and previous surgery – presented with cardiogenic shock following an out-of-hospital cardiac arrest. Resuscitation and emergency coronary angioplasty were successfully implemented, followed by admission to the intensive care unit. Antiplatelet therapy was started with aspirin 75 mg per day. Because of tachyarrhythmia-related cardiovascular compromise, the patient was placed on veno-arterial extracorporeal life support (VA-ECLS). However, the acute onset of pulmonary edema required placement of a LAVD (Impella® 5.0; Abiomed Inc.; Danvers, MA, USA). A brain computed tomography (CT) scan following left-sided seizure onset revealed a right cerebellar infarct. After approximately two weeks, Impella® 5.0 was removed and successfully replaced by a centrifugal continuous-flow ventricular assist device (HVAD®; HeartWare®, Medtronic, Minneapolis, MN, USA). Aspirin dosing was not tapered off because of the previous coronary angioplasty. Seven hours after recovery from anesthesia, acute neurological deficits suggestive of IS (left hemiparesis; Glasgow Coma Scale score: 10) were evident. The patient was intubated and verbal responses were not assessable. After consultation with neurologists, endovascular treatment was deemed unfeasible because of delayed discovery of the neurological presentation. A CT scan performed at 20 h post-admission revealed a complete right sylvian IS with significant mass effect resulting in a midline shift of 4.8 mm in the absence of haemorrhagic transformation (Fig. 1). In light of the rapidly deteriorating neurological conditions and imaging evidence of IS, emergency DHC was performed after weighing surgical-related risks against potential benefits (Figs. 2 and 3). Aspirin 75 mg/day was stopped the day before surgery and no intraoperative prothrombotic medication was given. The pre-operative laboratory coagulation status was as follows: international normalized ratio (INR), 1.34; activated partial thromboplastin time (aPTT) ratio, 1.2; platelet count: 367,000/μL. Platelet function tests were not performed albeit expected to be impaired. Previous antiplatelet therapy did not technically affect emergency DHC. Anticoagulation (continuous infusion of unfractionated heparin at a dose of 25,000 UI/mL) was started by consensus of neurosurgeons and cardiac surgeons on the second post-DHC day. Unfractionated heparin infusion was titrated to achieve a target aPTT ratio of 1.2 for the first week and 1.8–2 for two subsequent weeks. Antiplatelet treatment was reintroduced two weeks after emergency DHC. Finally, unfractionated heparin infusion was titrated up to obtain an optimal aPTT ratio of 2.5–3. On the third post-DHC week, the patient was switched to oral anticoagulation. During the first two post-DHC weeks, the patient received empirical therapy with levetiracetam to prevent potential seizures elicited by brain edema.

Upon recovery from anesthesia after DHC, the patient was conscious and responsive to commands. Although left-side paralysis initially persisted, there was a gradual recovery in neurological symptoms that allowed transfer to a multidisciplinary rehabilitation center. Cardiac flows remained stable under control of the HVAD® device. The patient was referred for HT at five months from DHC, followed by cranioplasty one month thereafter. Both intra- and post-operative courses were uneventful.

## Discussion and conclusions

We describe a case of malignant IS following the implantation of a HVAD® device who was successfully treated with emergency DHC. Cestari et al. [1] have previously shown the clinical utility of LVADs for improving survival of patients with advanced HF. The reported prevalence of IS following LVAD implantation vary widely from 2 to 14% [2]. Unfortunately, early identification of neurological deterioration may be challenging owing to the prolonged post-operative clinical course. Although brain edema in the context of a LVAD-related stroke generally portends a poor prognosis [3], emergency DHC in our patient was successful and allowed regaining an acceptable neurological function before HT. Both size and location of DHC affect the treatment effect. Even if DHC was quite small it allowed for the patient good functional results. A previous study in the general population demonstrated that implementation of DHC within the first 24 h of an IS can lead to successful outcomes in young patients without low preoperative Glasgow coma scale scores [4]. However, the question as to whether these findings are applicable to patients implanted with a LVAD has not been previously addressed.

Patients with a history of LVAD implantation who develop an IS may be continued on anticoagulation and antiplatelet treatment. In our case, decisions on anticoagulation management were taken by consensus within an interdisciplinary team of cardiac surgeons, neurosurgeons, neurovascular physicians, and anesthesiologists. Our patient underwent emergency DHC following a temporary two-day interruption of previous anticoagulation therapy. Low-dose anticoagulation with continuous infusion of unfractionated heparin was started in the post-DHC phase, followed by the reintroduction of antiplatelet therapy. We are unaware of guidelines or standard protocols for implementing anticoagulation or antiplatelet therapy in patients implanted with a LVAD who had undergone neurosurgery. In this scenario, pump thrombosis should be aggressively prevented [5] through a careful monitoring of pump speed while preserving aortic ejection and maintaining an optimal blood pressure control. LVAD failure did not occur in our case and – possibly because of his young age coupled with optimized anticoagulation management – the patient’s neurological status progressively improved, being finally referred for HT at five months after DHC.

In light of our patient’s successful clinical course, DHC seems to be possible in young patients who develop IS after LVAD placement. An optimized anticoagulation management and collaborative team-based practice are paramount to ensure positive outcomes in such highly complex cases. Despite these good results this type of handling might merit further investigation in a higher study setting.

## Data Availability

Not applicable. All data supporting the conclusions are presented in the manuscript.

## Abbreviations

aPTT: Activated partial thromboplastin time
CT: Computed tomography
DHC: Decompressive hemicraniectomy
HF: Heart failure
HT: Heart transplantation
IS: Ischemic stroke
LVAD: Left ventricular assist device
VA-ECLS: Veno-arterial extracorporeal life support
